# Case report: Resuscitative endovascular balloon occlusion after iatrogenic injury of the common iliac artery during neurosurgical dorsal lumbar microdiscectomy

**DOI:** 10.3389/fmed.2023.1112847

**Published:** 2023-02-02

**Authors:** Mascha O. Fiedler, Dittmar Böckler, Henrik Giese, Erik Popp, Felix C. F. Schmitt, Markus A. Weigand, Philipp Erhart

**Affiliations:** ^1^Department of Anesthesiology, Heidelberg University Hospital, Heidelberg, Germany; ^2^Department of Vascular and Endovascular Surgery, Heidelberg University Hospital, Heidelberg, Germany; ^3^Department of Neurosurgery, Heidelberg University Hospital, Heidelberg, Germany

**Keywords:** REBOA, iatrogenic bleeding, lumbar spine surgery, endovascular aortic occlusion, case report

## Abstract

**Introduction and importance:**

This case report describes resuscitative endovascular balloon occlusion (REBOA) of the aorta in a patient with life-threatening iatrogenic bleeding of the right common iliac artery during elective dorsal lumbar spine surgery. REBOA is an emergency procedure for temporary intra-aortic balloon occlusion being increasingly reported and published since its inauguration in 1954. The interdisciplinary management of hemorrhage and technical notes for a successful REBOA procedure will be presented.

**Case presentation:**

A 53-year-old female patient was admitted to the neurosurgery clinic suffering from left-sided L5 radiculopathy. During surgery, the anterior longitudinal ligament was perforated and an arterial vessel was lacerated. The patient became hemodynamically unstable demanding prompt supine repositioning and cardiopulmonary resuscitation (CPR). REBOA enabled cardiovascular stabilization after 90 min of CPR and laparotomy with vascular reconstruction and contributed to the survival of the patient without major clinical deficits. The patient was discharged from the ICU after 7 days.

**Clinical discussion:**

Resuscitative endovascular balloon occlusion of the aorta is an emergency procedure to control life-threatening hemorrhage. REBOA should be available on-scene and applied by well-trained vascular surgery personnel to control vascular complications or extend to emergency laparotomy and thoracotomy with aortic cross-clamping in case of in-hospital non-controllable hemorrhages. In case of ongoing CPR, we recommend surgical groin incision, open puncture of the pulseless common femoral artery, and aortic balloon inflation in REBOA zone I. Hereby, fast access and CPR optimization for heart and brain perfusion are maintained.

**Conclusion:**

Training for REBOA is the decisive factor to control selected cases of in-house and outpatient massive arterial abdominal bleeding complications.

## 1. Introduction

Resuscitative endovascular balloon occlusion of the aorta (REBOA) is an emergency procedure for temporary intra-aortic balloon occlusion being increasingly reported and published since its inauguration by Hughes (1). The objective is to achieve hemodynamic stabilization, reduction in blood loss, and consequently to improve cerebral and coronary perfusion in cases of life-threatening traumatic and non-traumatic hemorrhages. REBOA procedures carrying life-threatening risks such as arterial access complications, organ ischemia, vascular trauma, and time loss (2) should be critically evaluated in addition to resuscitative laparotomy, thoracotomy with aortic cross-clamping, or manual aortic compression.

Iatrogenic vascular injury is an unusual (0.02–0.06%) fatal complication of orthopedic or neurosurgical lumbar-disk procedures (3) demanding immediate availability of a trained multidisciplinary team of emergency specialists and vascular and endovascular surgeons. This study is in accordance with the SCARE 2020 criteria (4).

## 2. Presentation of case

A 53-year-old female patient American Society of Anesthesioloy (ASA) I classified without relevant comorbidities or medication was admitted to the neurosurgery clinic suffering from left-sided L5 radiculopathy. She presented with left foot and big toe elevator paresis. The MRI scan showed a mediolateral disc herniation at the level of L4/5 compressing the left L5 nerve root. Unilateral interlaminar fenestration with microdiscectomy at the level of L4/5 was performed. During surgery, the anterior longitudinal ligament was accidentally perforated and an arterial vessel was lacerated. The patient became hemodynamically unstable demanding prompt supine repositioning and cardiopulmonary resuscitation (CPR). The initial rhythm was a pulseless electrical activity. CPR started at 7:10 p.m. The abdomen appeared tense and massive arterial bleeding was assumed. The vascular surgery team was alerted. Under persistent CPR, the left groin was opened by a specialized vascular surgeon and the left common femoral artery was exposed within 7 min at 07:35 p.m. After the puncture and introduction of a short 6Fr sheath, a 0.035-inch wire (Glidewire Advantage^®^ Terumo Medical Corporation, Tokyo, Japan) was introduced without the availability of X-ray fluoroscopy. The 6Fr sheath was replaced over the wire by a 90-cm long, 12-French sheath (Destination, Terumo) using the Seldinger technique. The length from the groin to the thoracic aorta was assessed *ex situ* and a Reliant™ stent graft balloon catheter (Medtronic, Santa Rosa, CA, USA), used routinely for intraoperative temporary aortic occlusion in rAAA, was introduced and placed at the level of the proximal thoracic aorta (REBOA zone I). Transesophageal echocardiography confirmed adequate positioning of the wire, sheath, and total balloon occlusion within the thoracic aorta. Balloon occlusion at 07:40 p.m. resulted in a bleeding control but cardiovascular arrest with brief periods of sinus rhythm persisted. A sustained resuscitation situation developed and the patient developed ventricular fibrillation. After three defibrillations, a return of spontaneous circulation (ROSC) was finally achieved at around 8:40 p.m.

Massive transfusion (in total: 13 red cell concentrates, 5 fresh frozen plasma, and 4 platelet concentrates) and the clinical shock protocol (5, 6) were simultaneously initiated. Balloon occlusion was sporadically released in agreement with the anesthetist in order to maintain arterial perfusion of the renal and visceral arteries. Intermitted chest compression was necessary to stabilize the patient under aortic balloon occlusion periods. The cumulative time of CPR was 90 min. Meanwhile, a shock catheter into the right internal jugular vein, radial arterial cannulation, and a central venous catheter into the left internal jugular vein were established. After further stabilization, CPR could be terminated and laparotomy was performed at 08:45 p.m. with the inflated aortic balloon in place. Manual compression and cross-clamping of the infrarenal aorta leads to additional bleeding control and detection of active bleeding from the right common iliac artery (CIA). The vessel perforation was sutured after selective clamping of the CIA and blood supply to the right lower limb was restored. The abdomen was temporally packed and closed by V.A.C.^®^ therapy.

**FIGURE 1:**
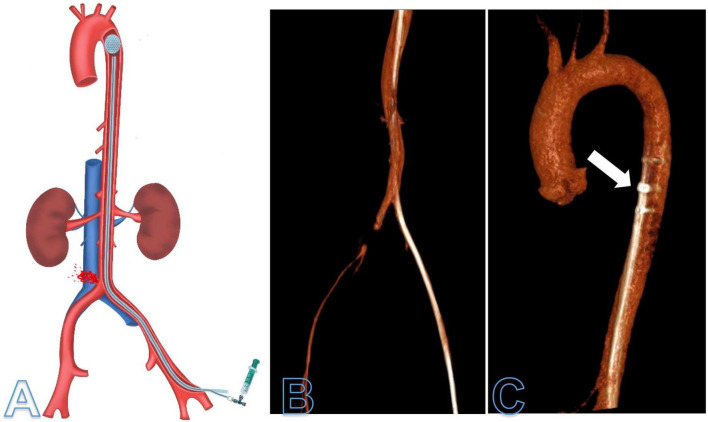
Illustration **(A)** and computed tomography angiography (CTA) reconstruction **(B,C)** of the deflated resuscitative endovascular balloon occlusion (REBOA) balloon in REBOA zone I **(C)**. CTA was performed after surgical bleeding control of the right common iliac artery was achieved. The 12Fr sheath and deflated aortic balloon were kept within the thoracic aorta (arrow) to enable additional aortic occlusion at any time during diagnostics.

Cranial, thoracic, and abdominal computed tomography (CT) angiography was performed to rule out irreversible complications. Despite restoring sufficient cardiac ejection function under ongoing massive transfusion, the 12Fr sheath and the deflated aortic balloon catheter were left in place to enable further aortic occlusion if necessary. An additional arterial blood pressure measurement was established during the CT diagnostic *via* the 12Fr sheath. Blood pressure was equal between the sheath and radial arterial blood pressure measurement. Cranial CT ruled out global brain ischemia. Figure 1 illustrates the CT imaging of the 12Fr sheath and the aortic balloon occlusion catheter at the level of the proximal thoracic aorta (REBOA zone I). Correct balloon positioning was confirmed and CT diagnostics excluded collateral damages after emergency REBOA intervention.

The abdomen was secondary closed and the sheath was removed from the left common femoral artery 3 h after the index procedure.

After intensive care stabilization, a secondary fasciotomy was necessary to decompress reperfusion compartment syndrome of the right calf. The initial left-sided foot drop weakness persisted, and a small asymptomatic subdural hematoma was demonstrated on a cranial follow-up CT. The patient survived without neurological sequela and was transferred to rehabilitation 3 weeks after surgery.

## 3. Discussion

This case report illustrates the importance of the trained interdisciplinary use of REBOA in life-threatening bleeding complications. REBOA should be available on-scene and applied by well-trained vascular surgery personnel to control vascular complications or extend to emergency laparotomy and thoracotomy with aortic cross-clamping in case of in-hospital non-controllable hemorrhages.

Resuscitative endovascular balloon occlusion of the aorta is an emergency procedure to control life-threatening hemorrhage. Side effects such as inadequate puncture attempts or inappropriate balloon insertions are considerably high (2).

The block balloon can be placed either in the infrarenal aorta (REBOA zone III) or in the descending aorta (REBOA zone I), depending on the indication or the suspected bleeding location (7). Aortic ballooning should be avoided in REBOA zone II to preserve gastrointestinal perfusion. In case of ongoing CPR, we recommend surgical groin incision, open puncture of the pulseless common femoral artery, and aortic balloon inflation in REBOA zone I. Based on bleeding location and imaging-guided vs. blind REBOA placement methods, different REBOA-related complications and mortality rates are described (8). Hereby, fast access and CPR optimization for heart and brain perfusion are maintained. As overall survival decreases after 30 min of continuous aortic balloon occlusion (9), partial-REBOA with temporary deflation periods is recommended to maintain blood supply to the visceral organs. From animal studies, it is presumed that ischemia-related mortality occurs after 60 min in REBOA zone 1 and after 90 min in REBOA zone III (10).

In the reported setting, a standard over-the-wire aortic balloon and long 12Fr sheath were used. Even though easily applicable REBOA balloons are available, we recommend over-the-wire placements and a long sheath for positioning control of the inflated balloon as pulse propagation may lead to unwanted migration of the REBOA balloon distally.

Whenever possible, REBOA should be performed under fluoroscopy in order to guarantee correct balloon positioning. In addition, fluoroscopy angiography can detect bleeding localization, and subsequently covered stent graft deployment may treat exsanguinations.

In a recently published “real world” case series of seven REBOA maneuvers, the time for REBOA establishment was 7–40 min (9). Fast decision-making and experience in vascular access strategies are essential. We recommend preparing and holding all REBOA materials available in emergency rooms (refer to Table 1). This case report encourages training in REBOA maneuvers.

**TABLE 1:** Resuscitative endovascular balloon occlusion (REBOA) sequences and material for an emergency over-the-wire in-house application.

Function	Material
Access to common femoral artery	Percutaneous: ultrasound guided puncture, 6 Fr sheath in seldinger technique Cutdown: scalpel, retractor, dissection set
Puncture and access to common femoral artery	6Fr sheath (short)
Wire insertion into thoracic aorta	0,035′′ wire (180 cm), e.g., Glidewire Advantage
Sheath replacement over the wire	12Fr sheath (long, 90 cm)
Balloon insertion and aortic occlusion	Aortic balloon (working length 100 cm, diameter 20–30 mm)
Additional material	Sterile coverage, syringe 20 ml, saline solution, suture material, and vascular clamps

Alternatively, specific REBOA catheters are available and can be used.

In this case, iliac artery injury was the cause of bleeding being a rare complication of lumbar spine surgery with life-threatening consequences.

## 4. Conclusion

Resuscitative endovascular balloon occlusion should be applicable and trained at any surgical center not only in the context of trauma care but also to control surgical in-house bleeding complications.

## Data availability statement

The original contributions presented in this study are included in this article/supplementary material, further inquiries can be directed to the corresponding author.

## Ethics statement

Ethical review and approval was not required for the study on human participants in accordance with the local legislation and institutional requirements. Written informed consent was obtained from the patient for publication of this case report and any accompanying images.

## Author contributions

MF, PE, and HG contributed to the study concept, data collection, data analysis, and writing the manuscript, and final editing of the manuscript. DB, MW, and EP revised the manuscript. MF, EP, FS, and PE approved the final version of the manuscript. MF was a guarantor. All authors contributed to the article and approved the submitted version.

## References

[1] Hughes C. Use of an intra-aortic balloon catheter tamponade for controlling intra-abdominal hemorrhage in man. *Surgery.* (1954) 36:65–8. 13178946

[2] Ribeiro Junior M Feng C Nguyen A Rodrigues V Bechara G de-Moura R The complications associated with Resuscitative Endovascular Balloon Occlusion of the Aorta (REBOA). *World J Emerg Surg.* (2018) 13:20. 10.1186/s13017-018-0181-6 29774048 PMC5948672

[3] Kwinta B Myszka A Bigaj M Dragan M Kenig J Krzyzewski R. Iatrogenic common iliac vessel injury during routine degenerative lumbar spine surgery: report of 2 cases and review of literature. *World Neurosurg.* (2020) 137:111–8. 10.1016/j.wneu.2020.01.168 32006736

[4] Agha R Franchi T Sohrabi C Mathew G Kerwan A Group S. The SCARE 2020 guideline: updating consensus surgical CAse REport (SCARE) guidelines. *Int J Surg.* (2020) 84:226–30. 33181358 10.1016/j.ijsu.2020.10.034

[5] Meneses E Boneva D McKenney M Elkbuli A. Massive transfusion protocol in adult trauma population. *Am J Emerg Med.* (2020) 38:2661–6. 10.1016/j.ajem.2020.07.041 33071074

[6] Spahn D Bouillon B Cerny V Duranteau J Filipescu D Hunt B The European guideline on management of major bleeding and coagulopathy following trauma: fifth edition. *Crit Care.* (2019) 23:98. 10.1186/s13054-019-2347-3 30917843 PMC6436241

[7] Wortmann M Engelhart M Elias K Popp E Zerwes S Hyhlik-Durr A. [Resuscitative endovascular balloon occlusion of the aorta (REBOA) : current aspects of material, indications and limits: an overview]. *Chirurg.* (2020) 91:934–42. 10.1007/s00104-020-01180-0 32514942 PMC7581582

[8] Matsumoto S Funabiki T Kazamaki T Orita T Sekine K Yamazaki M Placement accuracy of resuscitative endovascular occlusion balloon into the target zone with external measurement. *Trauma Surg Acute Care Open.* (2020) 5:e000443. 10.1136/tsaco-2020-000443 32426527 PMC7228664

[9] Johnson M Neff L Williams T DuBose J. Partial resuscitative balloon occlusion of the aorta (P-REBOA): Clinical technique and rationale. *J Trauma Acute Care Surg.* (2016) 81(5 Suppl 2):S133–7. 10.1097/TA.0000000000001146 27244578 PMC8789541

[10] Doucet J Coimbra R. REBOA: is it ready for prime time? *J Vasc Bras.* (2017) 16:1–3. 29930615 10.1590/1677-5449.030317 PMC5829684

